# Editorial: Personalized Medicine and Neurosurgery

**DOI:** 10.3389/fsurg.2017.00019

**Published:** 2017-04-10

**Authors:** M. Yashar S. Kalani, Nicholas Theodore

**Affiliations:** ^1^Department of Neurosurgery, Barrow Neurological Institute, St. Joseph’s Hospital and Medical Center, Phoenix, AZ, USA

**Keywords:** personalized medicine, editorial, neurosurgery, precision medicine initiative, contributions

**Editorial on the Research Topic**

**Personalized Medicine and Neurosurgery**

The Precision Medicine Initiative, which was instituted by President Barack Obama on January 20, 2015, highlighted the importance that advances in genomics and related “-omic” approaches have made to science and medicine, and it set the stage for their federally funded and mandated integration into the delivery of health care. Whether these advances comprise large-scale approaches, such as The Cancer Genome Atlas, which provides a modern classification of cancers based on molecular profiles, or genealogy initiatives, which seek to trace the movement of our early ancestors out of Africa, genomic technology has taken us closer to developing targeted therapies and a refined understanding of our evolutionary journey. It is against this backdrop that we summarized some of the recent advances in the field of precision, or personalized, medicine as they pertain to neurosurgery. In this e-Book collection provided by *Frontiers in Surgery: Neurosurgery*, we present a collection of 13 articles by leaders in the field of neurosurgery that highlight domains using a personalized approach for the treatment of patients or avenues when personalization is possible and when it will likely alter the care of patients with neurological diseases.

The contributions in this collection are broadly divided into three sections pertaining to vascular neurosurgery, oncology, and spinal disorders. In the realm of vascular neurosurgery, contributions from Fennell et al. summarize the biology of saccular aneurysms and avenues for development of new therapies for this disease process. Achrol and Steinberg continue the discourse by highlighting emerging therapies for subarachnoid hemorrhage and sequelae of aneurysm rupture, both areas where great advances are possible. Baranoski et al. review the emerging literature on cerebral cavernous malformations, a relatively common etiology of hemorrhagic stroke and one with treatment likely to be altered by the introduction of small molecule inhibitors targeting cavernous malformation formation.

The oncology collection is rich with both surgical and molecular advances that have made treatment of primary and metastatic tumors of the central nervous system safer and more effective. Ghinda and Duffau present their experience with intraoperative mapping for personalization of treatment of low-grade gliomas. Belykh et al. summarize the advances in intraoperative fluorescence imaging that have allowed for more aggressive and extensive resection of tumors in the brain. Razavi et al. provide a summary of mechanisms of immune evasion characteristic of glioblastoma multiforme. Bi et al. provide an authoritative review on the genomics of meningiomas, focusing on the diagnostic and prognostic implications of new information. Schunemann et al. summarize the state of the art in the management of von Hippel–Lindau disease. Mooney et al. and Hardesty and Nakaji complete the section with a review of pituitary adenoma biology and a review of the mechanisms of brain metastasis, respectively.

The section on spinal disorders includes a collection of papers by spinal neurosurgeons at Barrow Neurological Institute. Walker et al. review advances in biomarker development for spinal cord injury, and Martirosyan et al. and Martirosyan et al. describe novel findings on the biology of degenerative disc disease and the role of microRNA markers in predicting spinal cord injury.

With further advances in next-generation genomics technology and a decrease in its cost, physicians are likely to find that genomic information constitutes a pillar in their diagnostic and prognostic assessment of patients. Certainly, a much larger body of work focusing on other disease states could have been included in a collection such as this, but we sought to begin with a concise and select group of surgical neurological diseases. We hope to see our colleagues applying and taking the lead in the development of precision approaches to neurosurgical patients.

## Author Contributions

All authors listed have made substantial, direct, and intellectual contribution to the work and approved it for publication.

## Conflict of Interest Statement

The authors declare that the research was conducted in the absence of any commercial or financial relationships that could be construed as a potential conflict of interest.

